# Identification of plant based potential antifungal compounds against BMK-1 protein of *Bipolaris oryzae* using molecular docking approach

**DOI:** 10.1038/s41598-024-61431-6

**Published:** 2024-07-08

**Authors:** Sheeba Bhat, Mariya Rather, Saima Gani, Asha Nabi, Shabir Ahmad Ganai, Mehraj D. Shah, Parvaze Sofi, Fehim Jeelani, Arif Hussain, Sabiha Ashraf, Ali Anwar, Iram Iqbal, Tawkeer Un Nisa, Baby Summuna, Saba Banday

**Affiliations:** 1 Division of Plant Pathology, Faculty of Agriculture, Sher-e-Kashmir University of Agricultural Sciences and Technology-Kashmir, Wadura, Sopore, Jammu and Kashmir 193201 India; 2 Division of Plant Pathology, Faculty of Horticulture, Sher-e-Kashmir University of Agricultural Sciences and Technology-Kashmir, Shalimar, Srinagar, Jammu and Kashmir 190025 India; 3 Division of Genetics and Plant Breeding, Faculty of Agriculture, Sher-e-Kashmir University of Agricultural Sciences and Technology-Kashmir, Wadura, Sopore, Jammu and Kashmir 193201 India; 4 Division of Agri-Economics and Statistics, Faculty of Agriculture, Sher-e-Kashmir University of Agricultural Sciences and Technology-Kashmir, Wadura, Sopore, Jammu and Kashmir 193201 India; 5 Division of Horticulture, Faculty of Agriculture, Sher-e-Kashmir University of Agricultural Sciences and Technology-Kashmir, Wadura, Sopore, Jammu and Kashmir 193201 India; 6 College of Temperate Sericulture, Sher-e-Kashmir University of Agricultural Sciences and Technology-Kashmir, Mirgund, Jammu and Kashmir 193121 India; 7 Research Centre for Residue and Quality Analysis, Faculty of Horticulture, Sher-e-Kashmir University of Agricultural Sciences and Technology, Shalimar, Srinagar, Jammu and Kashmir 190025 India; 8 Directorate of Research, Faculty of Horticulture, Sher-e-Kashmir University of Agricultural Sciences and Technology of Kashmir, Shalimar, Srinagar, Jammu and Kashmir 190025 India

**Keywords:** *Bipolaris oryzae*, Clove, *Inula racemosa*, Noscapine, Plant extracts, Ursolic acid, Biological techniques, Plant sciences

## Abstract

Rice brown spot is an important disease of rice worldwide that inflicts substantial yield losses. The antimicrobial potential of methanol, acetone and dimethyl sulfoxide (DMSO) extracts of different medicinal plants, viz., *Syzygium aromaticum, Saussurea costus, Acorus calamus, Bergenia ciliate, Geranium pratense, Mentha longifolia, Inula racemosa, Podophyllum hexandrum, Heracleum candicans and Picrorhiza kurroa*, against the brown spot pathogen *Bipolaris oryzae *in vitro was evaluated via mycelial growth inhibition and spore germination inhibition assays. Among the plant extracts tested, 100% mycelial inhibition was observed for the methanol extract of *Syzygium aromaticum* at all three concentrations (2000 ppm, 3000 ppm and 4000 ppm), followed by the methanol extract of *Inula racemosa* (90.33%) at 4000 ppm. A maximum conidial germination inhibition of 83.54% was exhibited by the *Heracleum candicans* leaf extract. Phytochemical profiling of *Syzygium aromaticum* and *Inula racemosa* through liquid chromatography and mass spectrometry (HR-LCMS) revealed the presence of several compounds, such as eugenol, ursolic acid, quercetin, chlorogenic acid, and noscapine. A molecular docking approach was used to identify key inhibitory molecules against *B. oryzae*. Among the compounds detected in *S. aromaticum* and *Inula racemosa*, ursolic acid and noscapine were found to have the greatest binding affinity for the Big Mitogen Activated Protein Kinase (BMK-1) enzyme present in *B. oryzae*. In conclusion, *S. aromaticum* and *Inula racemosa* are potent compounds that could serve as lead compounds for drug discovery in the future.

## Introduction

Rice (*Oryza sativa* L.) is one of the most important cereal crops that feeds more than one-third of the world’s population^1^ and has the second-highest global production among agricultural commodities^2^. Approximately 90% of the rice grown in the world is produced and consumed in Asia, and India is the second largest producer of rice in the world.

Brown spot caused by *Bipolaris oryzae* (teleomorph = *Cochliobolus miyabeanus*) is a historically important disease of rice that occurs worldwide^3–5^ and causes substantial yield losses^6^. In India, the disease was reported to cause Bengal Famine in 1942, with 50–90% yield losses^7^.

Although chemical fungicides help in the management of this disease^8–10^, the continuous use of chemical fungicides leads to residual toxicity, environmental pollution and health hazards to humans and animals. In addition, the development of resistance in pathogens has raised serious concerns in plant disease management^11^. In this regard, there is a need to identify alternatives to existing chemical fungicides from time to time to manage the disease.

Plant-based products represent a valuable source of bioactive compounds with potent antimicrobial activities^12^. Recently, scientists have shown interest in identifying plant-based new antifungal compounds that are safe, ecofriendly and easily biodegradable^13^. Therefore, it would be advantageous to regulate different extraction solvents and in vitro antimicrobial efficacy testing so that the search for new bioactive compounds could be more systematic. Studies have been conducted to evaluate the antifungal activity of different plant extracts against *B. oryzae*^14^. However, the bioactive compounds from these botanicals have not been characterized. Currently, bioactive compounds can be characterized through new techniques such as high-resolution liquid chromatography‒mass spectrometry (HR-LCMS), gas chromatography‒mass spectrometry (GC-MS) or thin layer chromatography (TLC)^15^. In addition, molecular docking has emerged as a powerful computational tool for predicting the binding affinity of a compound for its target^16^. In silico assays coupled with bioassays may subsequently lead to the identification of plant-based compound(s) that could be used as alternatives to synthetic chemicals.

Historically, medicinal plants have been utilized for the treatment of various infectious and non-infectious diseases, and they continue to be a rich source of novel lead compounds and therapeutic agents. Natural products are rich sources of important drugs^17^. Plant-derived natural products are possible sources of novel therapeutic medicines for the future. In plant-based drug discovery and development research, plant metabolites are being optimized for developing potential analogues that can demonstrate the desired safety and efficacy. Technological developments have enabled the exploration of the profiles of complex phytoconstituents, leading to the isolation or synthesis of a number of successful therapeutic drugs and novel lead compounds that can provide a core scaffold for future drugs. It is particularly important to adopt modern drug development tools to accelerate the process of developing novel plant-origin therapeutic drugs. Therefore, the main aim of this study was to (i) determine the antifungal activity of various medicinal plant extracts against *Bipolaris oryzae* via comparison of extraction solvents and (ii) identify potential antifungal compounds (s) in the most potent plant extracts.

## Results

### Identification of the pathogen

**Figure 1:**
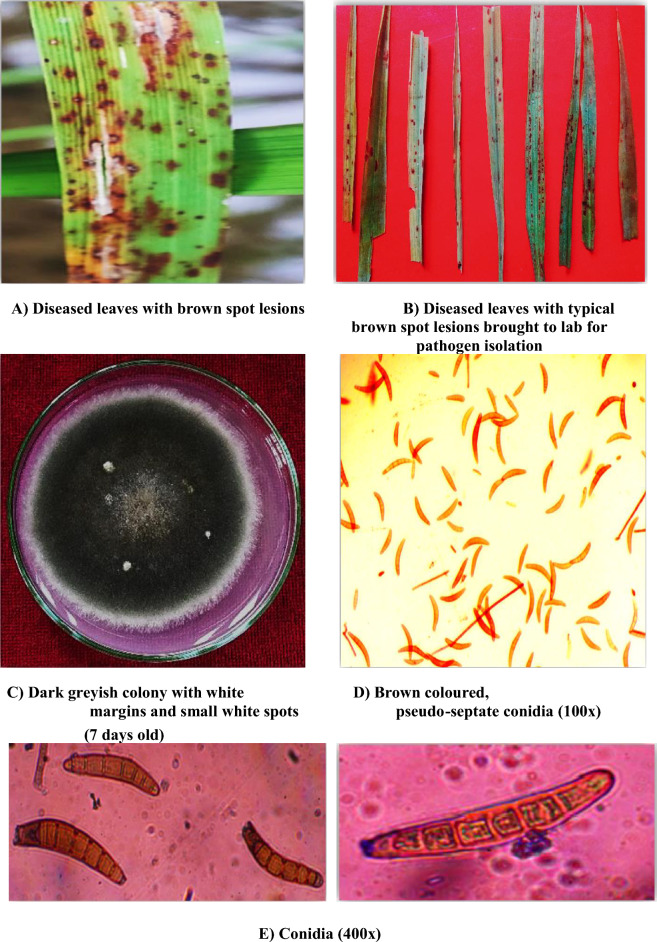
Brown spot symptoms, cultural and morphological characteristics of *Bipolaris oryzae.*

The colony of the fungus was composed of aerial as well as submerged mycelium and was grayish in color with white margins and white spots. The conidia were curved or slightly curved, fusiform, and light to dark brown in color. Pseudoseptation in conidia was observed, and the number of septa ranged from 4 to 10. The conidial size varied from 90.34 to 137.48 μm in length and 13.10 to 23.5 μm in width (Fig. 1).

### Antifungal activity assay of the plant extracts

#### In vitro evaluation of the effect of plant extracts on the radial growth of *Bipolaris oryzae*

##### Effect of the methanol plant extracts on the mycelial growth of *Bipolaris oryzae*

**Figure 2:**
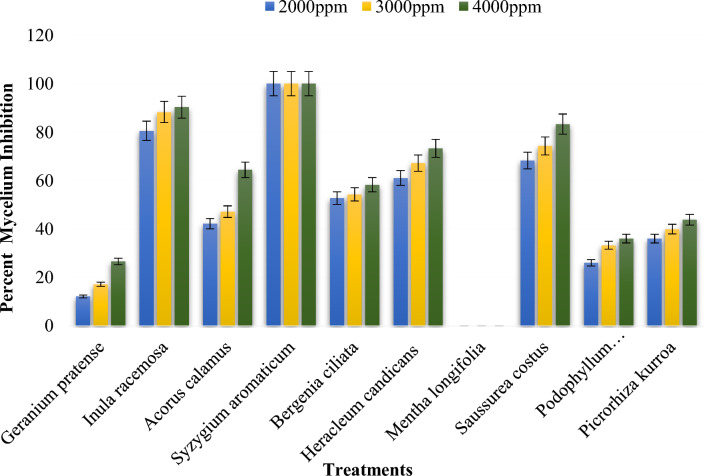
Percent mycelium inhibition shown by methanol plant extracts on *Bipolaris oryzae.*

**Figure 3:**
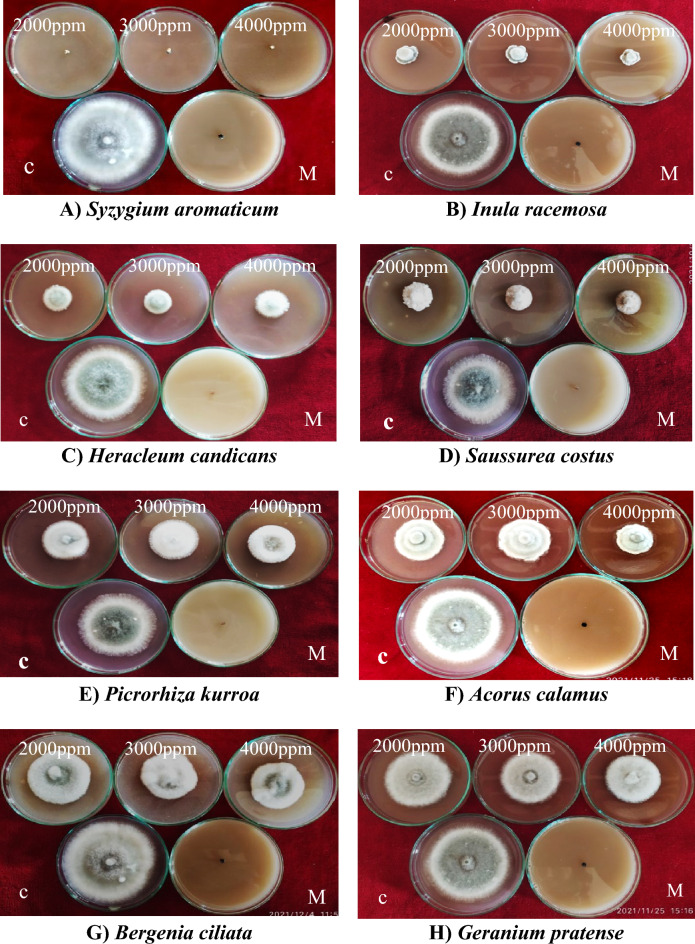
Response of *Bipolaris oryzae* to various methanol plant extracts. *C* Control, *M* Mancozeb.

The inhibition of mycelial growth of *Bipolaris oryzae* by various methanol plant extracts ranged from 0 to 100% (Supplementary Table 2). Among the plant extracts evaluated, the greatest inhibition of mycelial growth (100%) was observed in *Syzygium aromaticum* flower bud extract, followed by an inhibition of 90.33.40% for *Inula racemosa* root extract at 4000 ppm (Figs. 2, 3). The *Saussurea costus* root extract exhibited 75.33% inhibition, followed by the *Heracleum candicans* leaf extract (67.22%), the *Bergenia ciliata* root extract (55.14%) and the *Acorus calamus rhizome* extract (51.29%). There was 0.00% mycelial inhibition in the presence of the *Mentha longifolia* leaf extract. The mycelial inhibition of *Bipolaris oryzae* was significantly lower at 2000 ppm than at 3000 ppm and 4000 ppm (Fig. 2). The mean overall inhibition observed for the three concentrations (2000 ppm, 3000 ppm, and 4000 ppm) was 47.94, 52.20 and 57.64%, respectively (Supplementary Table 2). Furthermore, the methanol extract of clove flower buds (*Syzygium aromaticum*) was most effective at all three concentrations (2000 ppm, 3000 ppm and 4000 ppm), with an inhibition of 100% (Figs. 2, 3). This result was similar to the inhibition (100%) observed for the fungicide Mancozeb @ 1500 ppm, which was used as a standard control in this study.

##### Effect of acetone plant extracts on the mycelial growth of *Bipolaris oryzae*

**Figure 4:**
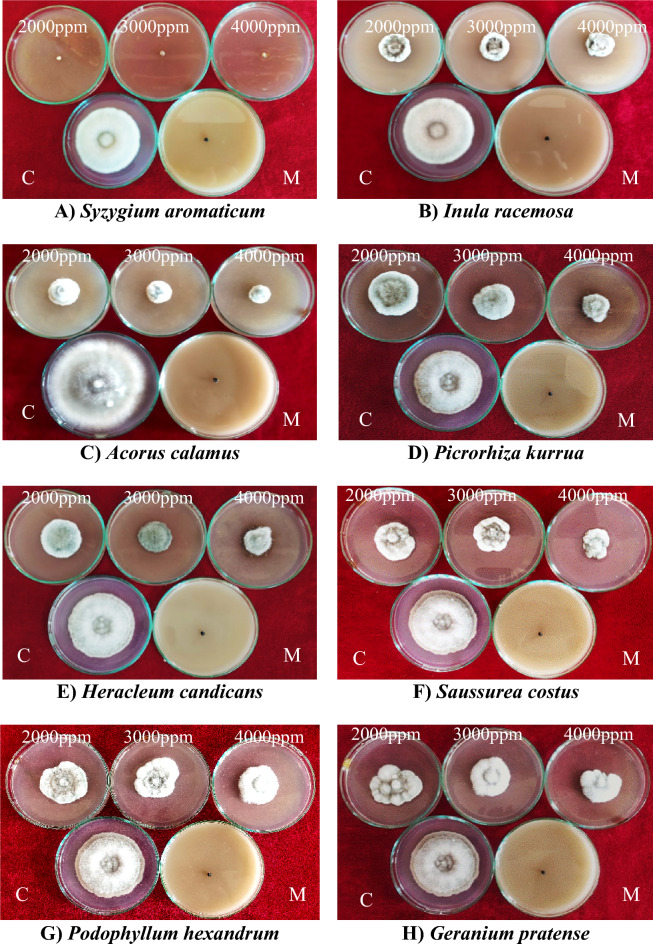
Response of *Bipolaris oryzae* to various acetone plant extracts. *C* Control, *M* Mancozeb.

**Figure 5:**
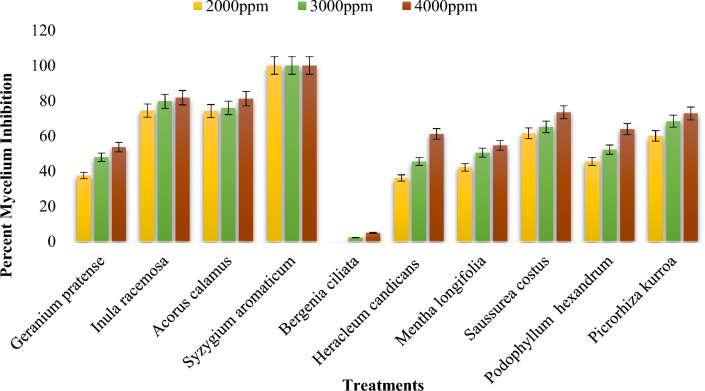
Percent mycelium inhibition shown by acetone plant extracts on *Bipolaris oryzae.*

The effect of acetone plant extracts on the radial growth of *Bipolaris oryzae* was significant (Supplementary Table 2). Among the ten acetone plant extracts tested at 4000 ppm, clove extract (*Syzygium aromaticum*) was the most effective, with a mycelial inhibition of 100% (Fig. 4), followed by *Inula racemosa* root extract (81.67%), *Acorus calamus* rhizome extract (81.14%), *Picrorhiza kurroa* stem extract (72.78%), *Saussurea costus* root extract (73.44%) and *Podophyllum hexandrum* root extract (63.89%) (Fig. 4). Minimum inhibition was observed in the case of the *Bergenia ciliata* root extract (5.00%). Furthermore, clove (*Syzygium aromaticum*) flower bud extract was the most effective at all concentrations tested (2000 ppm, 3000 ppm, and 4000 ppm), with a mycelial inhibition of 100%, which was similar to the inhibition (100%) observed for the fungicide Mancozeb@1500 ppm, which was used as a standard control in this study (Fig. 4). Furthermore, the effects of three different concentrations (2000 ppm, 3000 ppm, and 4000 ppm) on the mycelial inhibition of *Bipolaris oryzae* varied significantly, with mean overall inhibition values of 53.13, 58.75, and 64.74%, respectively (Fig. 5; Supplementary Table 2).

##### Effect of DMSO plant extracts on the mycelial growth of *Bipolaris oryzae*

**Figure 6:**
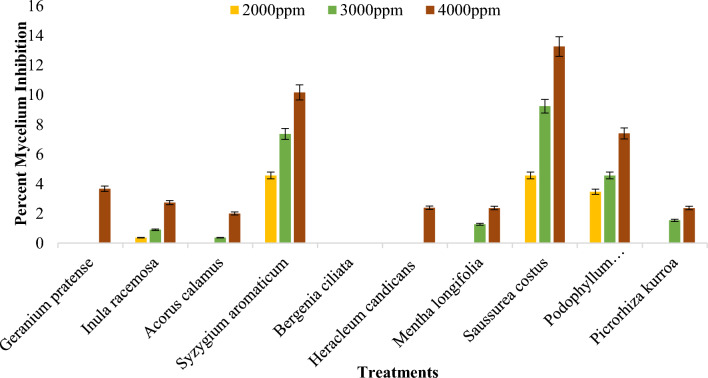
Percent mycelium inhibition shown by DMSO plant extracts on *Bipolaris oryzae.*

**Figure 7:**
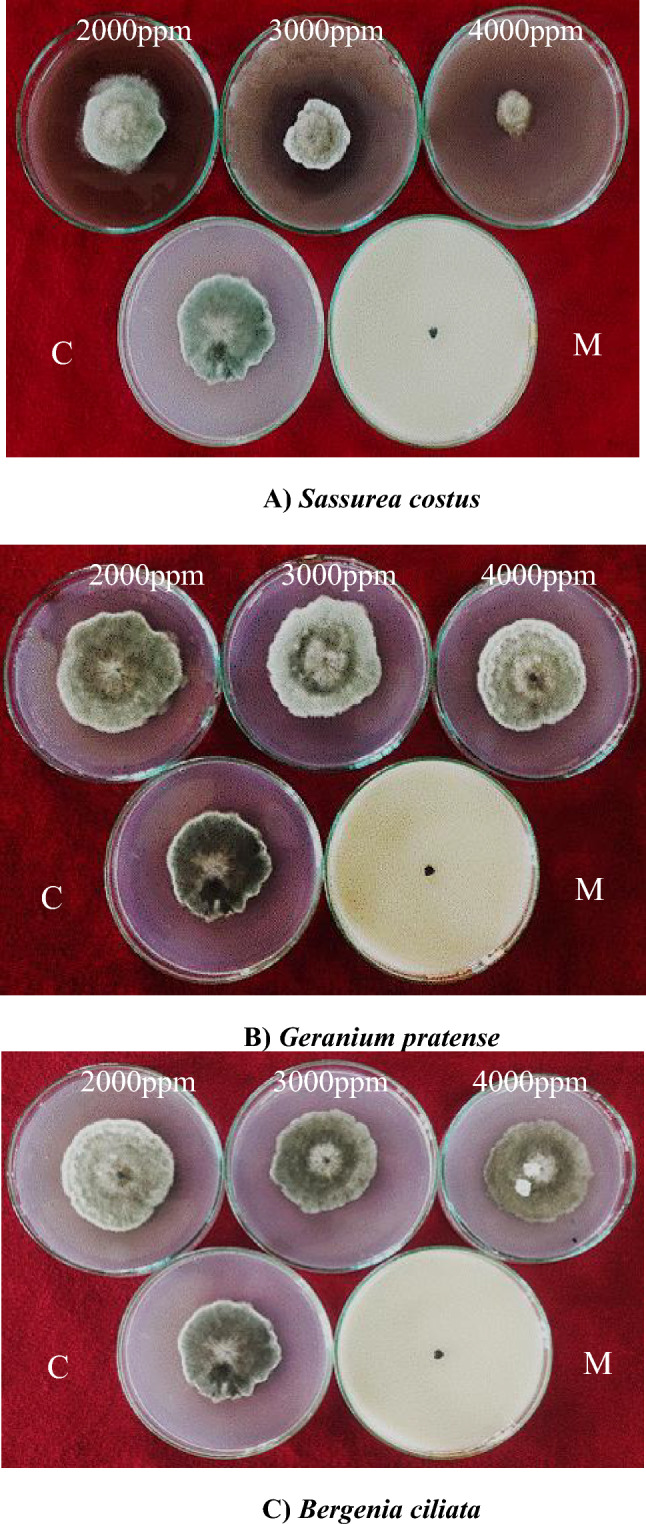
Response of *Bipolaris oryzae* to various DMSO plant extracts. *C* Control, *M* Mancozeb.

The inhibition of mycelial growth of *Bipolaris oryzae* by various DMSO plant extracts ranged from 0.00 to 9.02%, irrespective of the concentration (Supplementary Table 2). Among all the plant extracts tested at 4000 ppm, the maximum inhibition of mycelial growth (13.26%) over the control was observed for the *Saussurea costus* root extract (Fig. 7), followed by the *Syzygium aromaticum* bud extract (10.17%). The greatest mycelial inhibition was observed for the *Acorus calamus* rhizome extract (2.00%), *Heracleum candicans* leaf extract (2.38%) and *Bergenia ciliata* root extract (0.00%) (Figs. 6, 7).

##### Effect of the different extraction solvents on the antifungal activity of the plant extracts

Methanol, acetone and DMSO significantly inhibited the mycelial growth of *Bipolaris oryzae*. In this study, it was observed that the methanol and acetone extracts of the test plants exhibited better results than the DMSO extracts. The methanol and acetone extracts exhibited overall mean inhibition rates of 52.59% and 58.87%, respectively; however, DMSO showed a mean overall inhibition of 2.81% (Supplementary Table 2). Significant differences in the inhibition of conidial germination were detected between the methanol, acetone and DMSO extracts of the test plants (Supplementary Table 3). The methanol extracts exhibited the highest efficacy, with a mean overall spore germination inhibition of 62.98%, followed by the acetone extracts, with a spore germination inhibition of 61.44%; however, the DMSO extracts had the least effect, with an inhibition of 7.08% (Supplementary Table 3).

### In vitro evaluation of the effect of the plant extracts on the spore germination of *Bipolaris oryzae*

#### Effects of methanol plant extracts on the spore germination of *Bipolaris oryzae*

**Figure 8:**
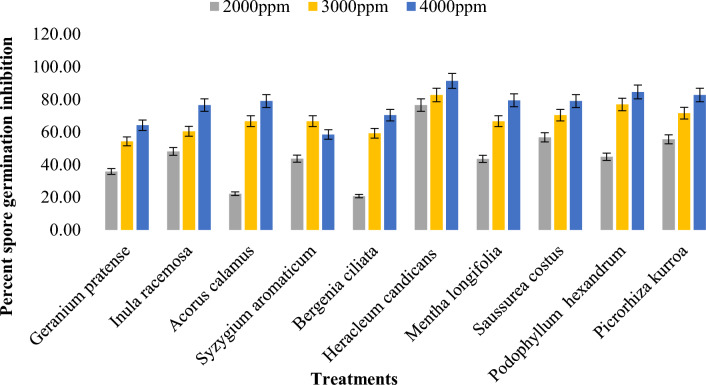
Effect of different concentration of methanol plant extracts on percent spore germination inhibition of *Bipolaris oryzae.*

The methanol plant extracts had a significant effect on the spore germination of *Bipolaris oryzae* (Supplementary Table 3). Among the ten methanol plant extracts tested at 4000 ppm, *Heracleum candicans* leaf extract exhibited the greatest inhibition of conidial germination (91.36%), followed by *Podophyllum hexandrum* (84.62%), *Picrorhiza kurroa* (82.72%) and *Mentha longifolia* leaf extracts (79.49%). *The effect of the Kenenia ciliata* root extract was the weakest, with an average spore germination inhibition of 50%. Furthermore, the effect of three concentrations (2000 ppm, 3000 ppm and 4000 ppm) on the conidial germination of *Bipolaris oryzae* varied significantly, i.e., with increasing concentrations from 2000 to 4000 ppm, the inhibition of spore germination of the pathogen also increased (Fig. 8). The mean overall inhibition at 2000 ppm, 3000 ppm and 4000 ppm was 44.80, 67.57 and 76.58%, respectively (Supplementary Table 3).

#### Effects of acetone plant extracts on the spore germination of *Bipolaris oryzae*

**Figure 9:**
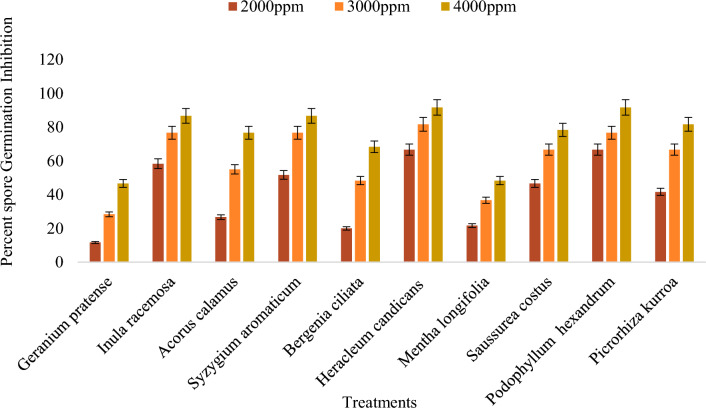
Effect of different concentration of acetone plant extracts on percent spore germination inhibition of *Bipolaris oryzae.*

All the acetone plant extracts had significant effects on spore germination in *Bipolaris oryzae* (Supplementary Table 3). Among the ten acetone plant extracts tested at 4000 ppm, *Heracleum candicans* leaf extract (91.67%) and *Podophyllum hexandrum* root extract (91.67%) had the greatest inhibition of spore germination, followed by *Inula racemosa* root extract (86.67%), *Syzygium aromaticum* bud extract (86.67%), and *Syzygium aromaticum* bud extract (48.16%). The whole plant extract of *G. pratense* had the least effect, with an average spore germination inhibition of 46.67%. With increasing concentrations from 2000 to 4000 ppm, there was an increase in the inhibition of pathogen germination (Fig. 9). The overall mean inhibition observed for the three concentrations, i.e., 2000 ppm, 3000 ppm and 4000 ppm, was 41.17, 61.34 and 75.67%, respectively (Supplementary Table 3).

#### Effects of the DMSO plant extracts on the spore germination of *Bipolaris oryzae*

**Figure 10:**
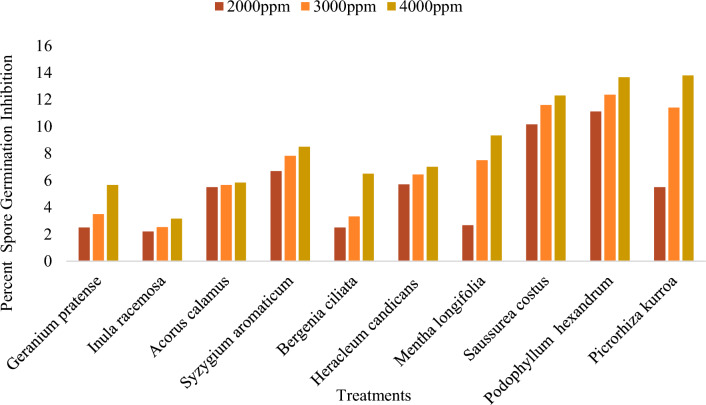
Effect of different concentration of DMSO plant extracts on percent spore germination inhibition of *Bipolaris oryzae.*

The results regarding the effect of the DMSO extracts of different test plants tested at 4000 ppm presented in the Supplementary Materials (Table 3) revealed that the greatest inhibition of conidial germination was exhibited by the *Picrorhiza kurroa* stem extract (13.80%), followed by the *Podophyllum hexandrum* root extract (13.67%). The minimum inhibition of conidial germination was observed for the *Inula racemosa* root extract (3.17%) (Fig. 10).

### Phytochemical profiling of the effective plant extracts

The findings of the in vitro antifungal bioassay of the selected plant extracts in this study showed that the methanol extracts of *Syzygium aromaticum* and *Inula racemosa* were the most effective at inhibiting the mycelial growth of *Bipolaris oryzae*. These extracts were subjected to LC‒MS to identify their bioactivity. The phytoconstituents detected in *Syzygium aromaticum* included polyphenols such as eugenol, gallic acid, and chlorogenic acid and specific flavonoids such as quercetin (Supplementary Table 4). In the case of *Inula racemosa*, the compounds identified were noscapine, quinic acid, cryptochlorogenic acid, caffeic acid and m-coumaric acid (Supplementary Table 5).

### Molecular docking

**Figure 11:**
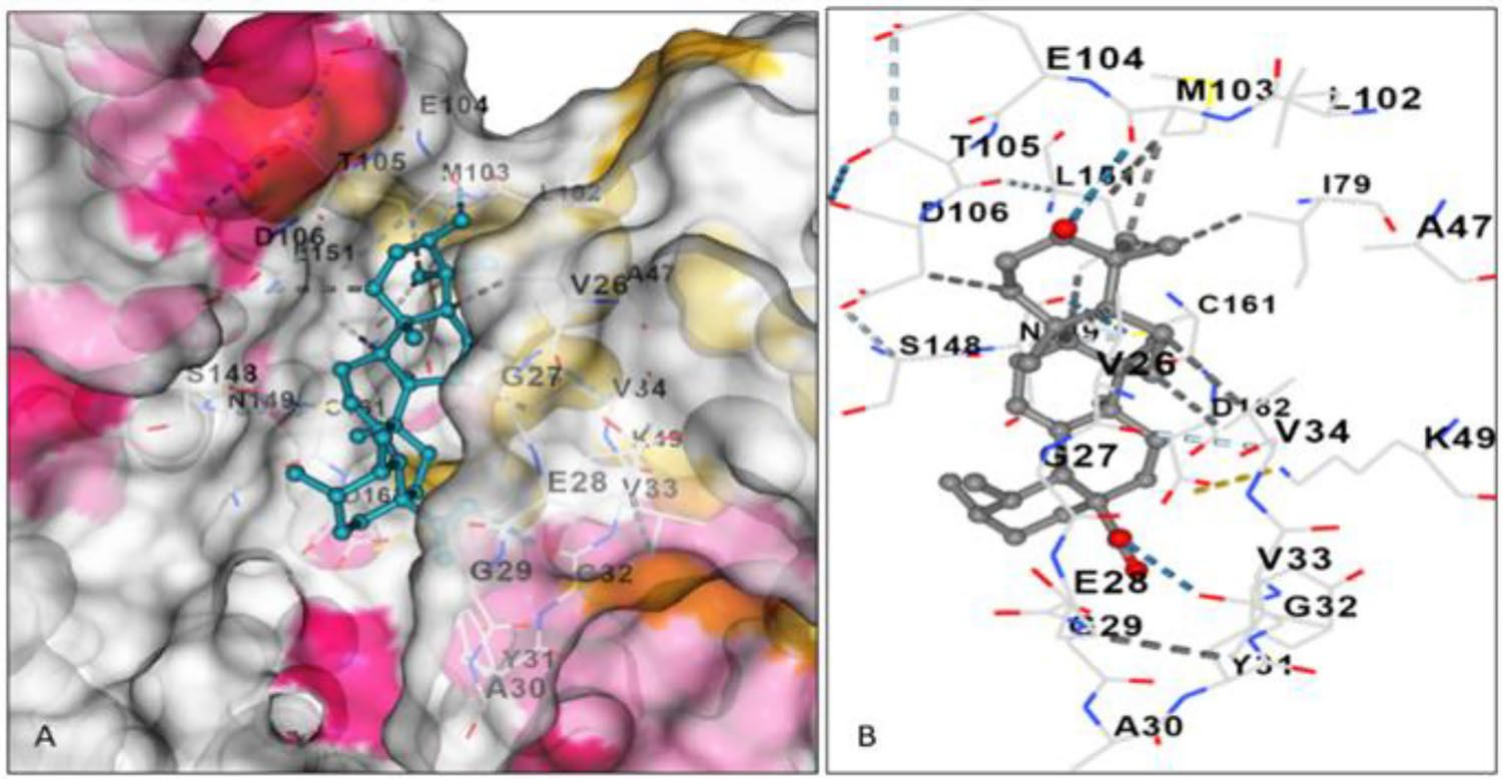
Ursolic acid in active site of BMK-1 (**A**). Active site amino acid residues of BMK-1 interacting with ursolic acid through non-covalent interactions (**B**).

**Figure 12:**
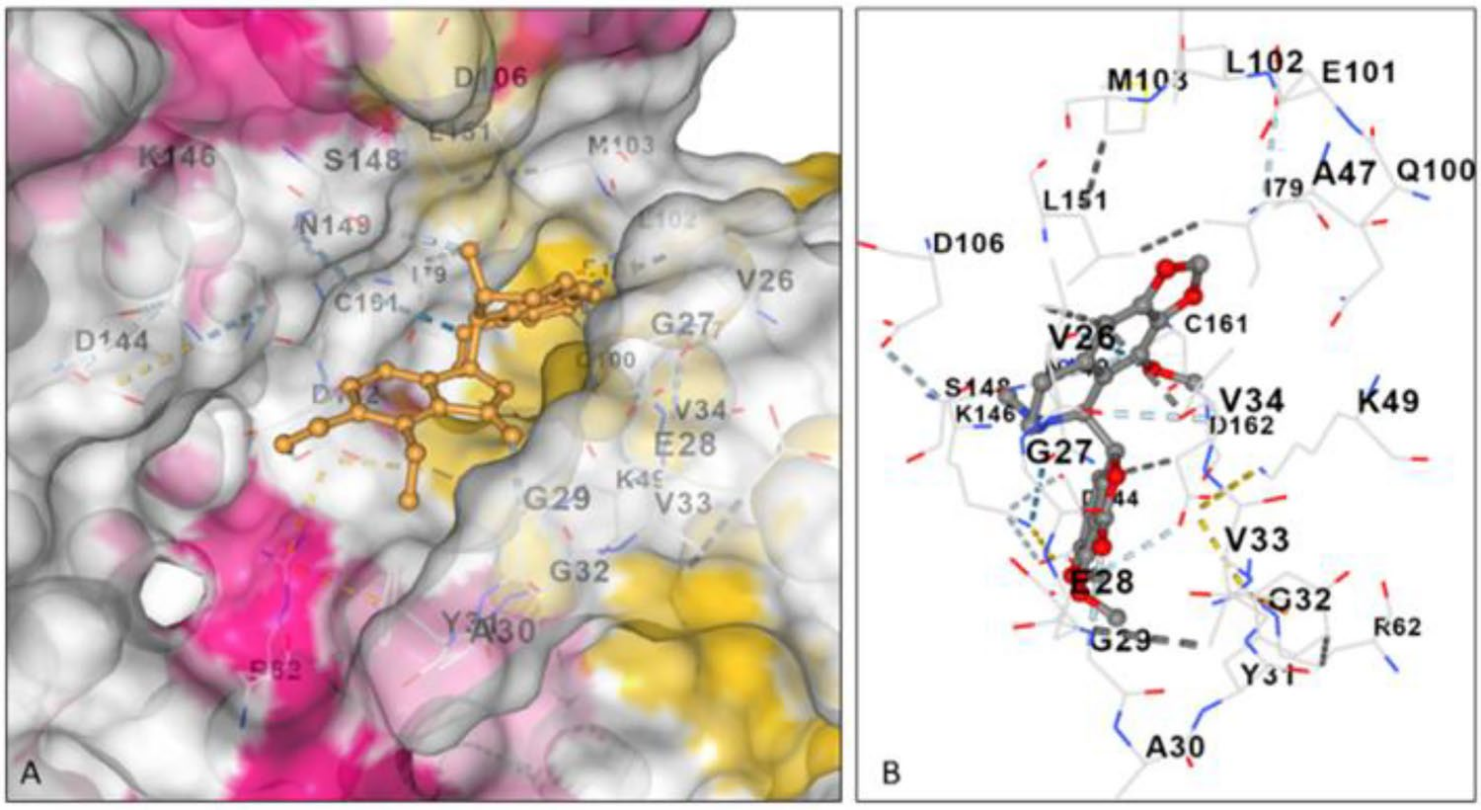
Noscapine in binding pocket of BMK-1 (**A**). Amino acid residues of BMK-1 active site interacting with noscapine (**B**).

To identify the main inhibitor compounds, the selected phytochemicals (chlorogenic acid, eugenol, gallic acid, quercetin, and ursolic acid) were docked with Big Mitogen Activated Proteinase Kinase-1 (BMK-1), an important enzyme responsible for growth and other conditions. The model quality (of the chosen model as described in the Materials and Methods section) proved to be appropriate for subsequent studies. The model revealed 81.3% residues in most favored regions, 15.8% in additional allowed regions and only 1.9% and 0.9% residues in generously allowed and disallowed regions, respectively^18^. Among the five molecules identified from clove, ursolic acid showed the greatest binding affinity, as indicated by its greater negative value (− 8.7 kcal/mol). This was followed by quercetin, chlorogenic acid, eugenol and gallic acid, as indicated by the binding energy values of − 8.1, − 8.0, − 5.2 and − 5.0, respectively (Supplementary Table 6). It should be noted that the lowest binding free energy indicates the highest affinity, and vice versa^19^. The maximum binding affinity of ursolic acid can be ascribed to its ability to form maximum interactions with the active site residues of BMK-1 (Supplementary Table 6; Fig. 11A,B)^20^. Among the molecules explored from *Inula racemosa*, noscapine demonstrated maximum affinity, as indicated by the binding free energy value of − 8.1 kcal/mol. After this, cryptochlorogenic acid was observed to have a maximum affinity of − 7.6, followed by m-coumaric acid (− 5.6), caffeic acid and quinic acid (− 5.2 and − 5.1). The highest binding affinity was shown for noscapine, which exhibited the most intense interaction profile, as it interacted with the maximum number of amino acid residues in BMK-1 (Supplementary Table 7)^21^. The presence of noscapine in the active site of BMK-1 and the residues targeted by this molecule are summarized in Fig. 12A,B.

## Discussion

The present study was undertaken to assess the antifungal efficacy of ten different plant extracts against *Bipolaris oryzae* and to identify potential antifungal compounds in the most efficient plant extract. The pathogen was isolated via the tissue bit method and purified via the single-spore technique, and its morpho-cultural characteristics were studied. The colony of the fungus was composed of aerial as well as submerged mycelium and was grayish in color with white margins and white spots. The conidia were curved or slightly curved, fusiform and light to dark brown in color. Pseudoseptation in conidia was observed, and the number of septa ranged from 4 to 10. The conidial size varied from 90.34 to 137.48 μm in length and 13.10 to 23.5 μm in width. Similar morphological characteristics have been described for *Bipolaris oryzae* previously^22,23^.

In vitro assessment of the percentage of mycelial inhibition by the Poisoned Food Technique and conidial germination inhibition of the fungus was performed with each plant extract. Plant extracts exhibited varying antifungal potential against *Bipolaris oryzae*, possibly due to the extraction process, extraction solvent, plant origin, plant type, responsiveness of the test pathogen and concentration of the different plant extracts^24,25^. Among the plant extracts assessed, complete inhibition of mycelial growth (100%) over the control was observed in the presence of methanol and acetone (*Syzygium aromaticum* bud extract) at concentrations of 2000 ppm, 3000 ppm and 4000 ppm, comparable to the mycelial inhibition (100%) exhibited by the fungicide Mancozeb, which was used as a control in the present study. The results for *S. aromaticum* bud extract are in agreement with those of Madi et al*.*^26^, who reported 100% inhibition of the mycelial growth and conidial germination of *Curvularia lunata* and *Fusarium sacchari* using bud extracts of *S. aromaticum* at concentrations of 1000 ppm, 5000 ppm and 10,000 ppm. Adhikari et al.^27^ reported that methanol *S. aromaticum* extract was more effective than gentamycin against *Pseudomonas aeruginosa,* thus corroborating the present findings*.* The antimicrobial and antifungal effects of *S. aromaticum* extracts can be attributed to the presence of phenolic compounds, flavonoids, hydroxybenzoic acids and hydroxyphenyl propenes^28^. Aulifa et al*.*^29^ reported that the n-hexane extract of clove buds (*Syzygium aromaticum*) is a natural fungicide and could be an alternative fungicide for *Phytophthora palmivora*. Methanol *Inula racemosa* root extract inhibited 90.33% at 4000 ppm, and similar results were reported by Omezzine et al*.*^30^, who reported that the growth of *Trichoderma* spp. and *Fusarium* spp. was completely inhibited by the methanol extract of *Inula* spp. Thus, *Inula* could be an important source of phytochemical compounds necessary for developing environmentally safer antifungal products. An inhibition of 81.67% at 4000 ppm was exhibited by the acetone rhizome extract of *Acorus calamus*, which is consistent with the findings of Rawal et al.^31^, who reported that the greatest inhibition by the acetone rhizome extract of *Acorus calamus* at a 1000 mg/ml concentration against *Fusarium oxysporum f. sp*. *lycopersici*. The leaf extract of *Heracleum candicans* inhibited mycelia by 73.33% at 4000 ppm. In their study, Lingaraju and Sudarshana^32^ reported that the ethyl acetate extract of *Heracleum rigens* showed the greatest antifungal activity at a minimum inhibitory concentration (MIC) of 1.56 μg/ml against *Candida albicans.* The methanol extract of *Mentha longifolia* showed no inhibition in this study, which contradicts reports by Ali et al.^33^, who reported significant inhibition of various Aspergillus spp. by the methanol leaf extract of *Mentha piperita*. This finding indicates that the antifungal activity of a particular plant extract depends not only on the plant species but also on the pathogen tested.

With increasing concentrations from 2000 to 4000 ppm, there was an increase in the inhibition of mycelial growth of the pathogen. The mean overall mycelial inhibition calculated for the three concentrations (2000 ppm, 3000 ppm and 4000 ppm) was 34.12, 37.82 and 42.33%, respectively. These results are consistent with the findings of Parveen et al.^34^, who observed maximum inhibition of mycelial growth at higher concentrations of test plant extracts. Chabra et al.^35^ also corroborated our findings, as they reported the pronounced effect of the concentration of plant extracts on the mycelial growth of *Bipolaris oryzae.*

It has been revealed that acetone plant extracts showed greater mycelium inhibition than methanol extracts. Several other studies have also described acetone as an efficient extraction solvent compared to other solvents, such as hexane, dichloromethane, and methanol. This is because acetone dissolves various lipophilic and hydrophilic constituents of plant extracts and easily volatilizes afterwards^36–38^. Acetone is more efficient than ethanol or methanol for the extraction of phenolic compounds, saponins and antioxidants^39^. Abirami et al*.*^40^, in their study of the antifungal activities of acetone extracts of medicinal plants (*Acalypha indica*, *Allium sativum*, *Citrus limon* and *Lawsonia inermis*) against fungal pathogens (*Alternaria* spp., *Curvularia* spp., *Fusarium* spp., *Trichophyton* spp. and *Geotrichum* spp.) found that all the acetone plant extracts inhibited the test pathogens. However, DMSO had less of an effect than the other solvents used in the study, possibly because acetone and methanol are more effective at extracting and dissolving crude extracts from plant matrices due to their good miscibility and nontoxicity at higher concentrations compared to DMSO^41^.

Among all the extracts, *Heracleum candicans* had the greatest inhibition of spore germination (57.31%)*,* followed by *Podophyllum hexandrum* (51.36%) and *Saussurea costus* (48.91%). *Heracleum* spp. have broad antimicrobial, antioxidant, antiviral and cytotoxic properties^42^, which may be the reason that *Heracleum* spp. strongly inhibited spore germination of the test pathogen. *Syzygium aromaticum* bud extract inhibited 42.29% of the bacteria. Nagerabi et al.^43^ reported that *Syzygium aromaticum* bud extract markedly inhibited the germination of *Aspergillus niger* spores by 43–96%. The antifungal potency of the *Syzygium aromaticum* extract could be attributed to the presence of phenolic compounds, flavonoids, hydroxybenzoic acids and hydroxyphenyl propenes^28^. Rana et al*.*^44^ reported that the antifungal activity of *Syzygium aromaticum* oil extract against fungal pathogens was due to the presence of compounds, including eugenol, β-caryophyllene, eugenyl acetate and α-humulene. Rojas et al*.*^45^ reported that clove has antifungal and antimicrobial activity due to the presence of various compounds, including caffeic acid, eugenol, ellagic acid, ferulic acid, gallic acid, salicylic acid and quercetin. The overall means of spore germination inhibition for the three different concentrations (2000 ppm, 3000 ppm and 4000 ppm) were 30.47, 45.37 and 53.61%, respectively, which indicates that increasing the concentration of a particular test plant extract obviously increased the percentage of spore germination inhibition of the pathogen. Similarly, Parveen et al.^46^ reported similar results on the inhibition of sporulation of the test fungi (*Dreschslera* spp., *Aspergillus flavus*, *Aspergillus niger* and *Penicillium expansum*).

Among the tested solvents (acetone, DMSO, and methanol), methanol plant extracts had the greatest inhibitory effect on spore germination (62.98%), followed by acetone (61.44%), and DMSO plant extracts had the least inhibitory effect (7.08%). Gholamnezhad^47^ reported that methanol plant extracts of neem, fennel, lavender, thyme, pennyroyal, salvia and asafoetida showed greater inhibitory effects in terms of spore germination inhibition against *Botrytis cinerea*, which causes postharvest disease of apple, and Truong et al.^48^ identified methanol as the most effective solvent because it provides the highest extraction yield with high antimicrobial potential. According to Lezoul et al.^49^, the higher efficacy of methanol extracts could be ascribed to the fact that methanol extraction produces more flavonoids and polyphenols than acetone and ethanol.

Phytochemical screening of *Syzygium aromaticum* methanol extract using high-resolution liquid chromatography combined with mass spectrometry (HR-LCMS) revealed the presence of alkaloids, flavonoids, aldehydes, terpenoids, glycosides, ketones, tannins, steroids, carbohydrates and phenolic compounds. The phytoconstituents found were eugenol, gallic acid, chlorogenic acid, quercetin and ursolic acid. Our findings are in agreement with those of Adhikari et al.^27^, who reported that the methanol extract of clove had the greatest inhibitory effect on *Proteus mirabilis*, *Pseudomonas aeruginosa* and *Candida albicans* due to the presence of phytochemicals such as flavonoids, polyphenols and tannins. Using gas chromatography‒mass spectrometry (GC‒MS) analysis of clove oil, Ayoola et al.^50^ reported that the phytoconstituents in clove were caryophyllene, eugenol, eugenol acetate and alpha-humelene, with eugenol being the leading phytoconstituent. Our results are also consistent with those of Jimoh et al.^51^, who, in their study on the phyto-chemical screening of clove extract using gas chromatography with flame ionization detection (GC-FID), reported phyto-chemicals such as alkaloids, terpenoids, flavonoids, tannins, and aldehydes. The major group of compounds identified were phenolic compounds such as eugenol, luteolin, salicylic acid, and gallic acid. Similar results were reported by Kumar et al*.*^52^ in their study on the phyto-chemical screening of dichloromethane extracts of clove. In the case of the methanol root extract of *Inula racemosa*, the phytochemical screening revealed the presence of alkaloids, flavonoids, flavonols, diterpenoids, sesquiterpenoids, quassinoids, cyclic polyols and hydrocinnamic acids. Sharma et al.^53^ isolated compounds from the root extract of *Inula racemosa* and reported antifungal properties against various pathogenic fungi (*Aspergillus flavus*, *Aspergillus niger*, *Candida tropicalis*, *Candida albicans* and *Geotrichum candidum)* due to the presence of various compounds, including sesquiterpene lactone, alantolactone, alloalantolactone, isoalantolactone, alkaloids and flavonoids. Mohan and Gupta^54^ reported the efficacy of *Inula racemosa* root extract due to the presence of polyphenols and flavonoids in aqueous, ethanolic and aqueous-ethanolic root extracts of *Inula racemosa*.

Advanced bioinformatics tools have made it easier to predict the binding affinity of bioactive compounds for proteins that are important for pathogen infection. These tools have successfully been employed for in silico identification of potential phyto-fungicide candidates against *Magnaporthe oryzae*, a rice blast pathogen^55^. To identify the inhibitory molecules against Big Mitogen Activated Proteinase Kinase-1 (BMK-1) of *B. oryzae*, which is an important enzyme related to growth, conidiation and pathogenicity, five compounds (chlorogenic acid, eugenol, gallic acid, quercetin, and ursolic acid) from clove were selected for molecular docking based on their antimicrobial activity, as mentioned in the literature. Ursolic acid proved to be the most potent molecule in terms of binding affinity toward the enzyme. The newly identified molecule could thus serve as a potential next-generation plant-based drug against *Bipolaris oryzae* after further analysis of this molecule. Mahlo and Eloff^37^ identified ursolic acid as an antifungal compound from acetone leaf extracts of *Breonadia salicina* (Rubiaceae) against three plant fungal pathogens (*Penicillium expansum*, *P. janthinellum* and *P. digitatum*). Singh et al.^56^ also employed a molecular docking approach to determine the binding mode and affinity of different phyto-chemical compounds for lanosterol 14α- demethylase (the target protein). Stigmasterol and γ-sitosterol in their study were found to have the highest binding affinity for the receptor protein. Similarly, five compounds, viz. Quinic acid, m-coumaric acid, caffeic acid, crypto-chlorogenic acid and noscapine in methanol root extracts of *Inula racemosa* (based on reports of their antimicrobial activity) were selected for molecular docking against the target protein BMK-1. The most efficient compound in terms of maximum binding affinity toward the target molecule BMK-1 was noscapine. This is the first study regarding the effectiveness of *Inula racemosa* against *Bipolaris oryzae.* However, more in-depth analysis of the individual activity and mode of action of the identified compounds as powerful inhibitors is needed.

## Methods

### Isolation and identification of the pathogen

Pathogen was isolated from diseased paddy leaves showing typical brown leaf spot symptoms via the tissue-bit method^57^. From the expanding edges of lesions and healthy regions, infected leaf samples were cut into tiny pieces, surface sterilized for 30 s with 0.1% mercuric chloride and then rinsed three times in sterile distilled water. Surface-sterilized leaf fragments were then aseptically inoculated in Petri plates in a laminar-air-flow cabinet on solidified potato dextrose agar (PDA) media under aseptic conditions and incubated at 25 ± 1 °C. The pathogen was purified using a single-spore technique^58^ and identified based on cultural and morphological characteristics as described by Alcorn^59^.

### Collection of plant materials and preparation of acetone, dimethyl sulfoxide (DMSO) and methanol extracts

Plant material was procured from the Regional Cum Facilitation Centre (RCFC), North of National Medicinal Plants Board, which is located at the Faculty of Agriculture, Wadura. The center complies with relevant institutional, national, and international guidelines and legislation for the collection and cultivation of plants. The samples were further identified at the Centre for Biodiversity & Taxonomy, Department of Botany, University of Kashmir, Srinagar, Jammu & Kashmir, India. The details of the plant material used in this study are listed in supplementary Table 1. The plant parts were washed with water, rinsed, and then shade-dried. The dried plant parts were ground into powder using an electric blender and stored in a tight glass container for further use. To produce a suspension, 15 g of ground plant powder was added to 100 ml of sterile water in a conical flask. The flask was then firmly plugged. For enhanced phytochemical extraction, the suspension was incubated in a shaker incubator at 25 °C for 4 days. Thereafter, the suspension was passed through Whatman's No. 1 filter paper to obtain a clear filtrate. The filtrates were dried in a hot air oven at 60 °C until all the solvent evaporated^60^. The same procedure was adopted for extraction in all the solvents.

### Reconstitution of the plant extract

The extract was reconstituted by weighing two grams of dried extract and dissolving it in 10 ml of sterile distilled water to obtain a stock concentration of 20%, which was then kept in sterilized tubes in a refrigerator at 4 °C for future studies. From this stock concentration, the working concentrations, viz., 2000 ppm, 3000 ppm and 4000 ppm, were made using dilution formulae.

### Antifungal activity assay of the plant extracts

#### Determination of mycelial growth inhibition by the poisoned food technique

The antifungal potency of the plant extracts was tested against *B. oryzae *in vitro using the Poisoned Food Technique^61^. The required volume of each plant extract was obtained with a pipette from a stock solution at a concentration of 2,00,000 ppm and added to 50 ml of sterilized PDA media in conical flasks to achieve the requisite final concentrations of 2000 ppm, 3000 ppm and 4000 ppm. Under aseptic conditions (in laminar air flow), 20 ml of PDA poisoned with the plant extract was placed into sterile petri plates and allowed to solidify. For each treatment combination, 5 mm *B. oryzae* mycelial discs from a young culture (7 days old) were inoculated in the center of PDA plates with plant extracts aseptically**.** Control plates (without plant extract) were also maintained. Each treatment was replicated thrice in a completely randomized design (CRD). Mancozeb (1500 ppm) was also used for comparison. The inoculated Petri plates were incubated at 25 ± 1 °C, and radial mycelial growth was recorded after 6 days of inoculation. The efficacy of selected extracts was measured in terms of percent inhibition of mycelial growth^62^.

$${\text{I}}\hspace{0.17em}=\hspace{0.17em}\frac{{\text{C}}-{\text{T}}}{{\text{C}}} \times 100$$

where I is the percent inhibition; C is the colony diameter in the control plate; and T is the colony diameter in the treated plate.

### Determination of spore germination inhibition

To determine the percentage of spore germination inhibition, the slide germination technique was used^63^. The requisite concentrations of 2000 ppm, 3000 ppm and 4000 ppm were prepared using the desired amount of plant extracts from the stock solution. Moreover, from a young culture of the pathogen, a spore suspension containing approximately 2 × 10^6^ spores per ml was prepared. One drop of the final concentration of plant extracts was obtained using a dropper, placed on cavity slides and then allowed to dry. A drop of spore suspension was placed on the cavity slide after it had dried. Furthermore, a control was maintained. The whole set up was arranged in a 90 mm petri dish with moistened blotting paper at the bottom and incubated at 25 ± 1 °C overnight. Observations of spore germination were recorded after 12 h of incubation under a microscope. The experiment was conducted in a completely randomized design (CRD) with three replications for each treatment. In addition, 0.15% (1500 ppm) mancozeb was used as a positive control. The percent germination inhibition for each plant extract was estimated using Vincent's formula^62^ as mentioned above:

$${\text{I}}\hspace{0.17em}=\hspace{0.17em}\frac{{\text{C}}-{\text{T}}}{{\text{C}}} \times 100$$

where I is the Percent spore germination inhibition; C is the Number of spores germinated in the control group; and T is the Number of spores germinated in the treatment group.

### Identification of the bioactive compounds

The highly effective plant extracts obtained (*Syzygium aromaticum* and *Inula racemosa*) were subjected to high-resolution liquid chromatography-cum-mass spectrometry (HR-LCMS/MS) for identification of the various phytoconstituents present in the plant extracts, which was performed at The Indian Institute of Technology, Bombay. The analysis was performed with a UHPLC-PDA detector mass spectrophotometer (1290 Infinity UHPLC System, 1260 infinity nano HPLC with Chipcube, 6550 iFunnel Q-TOFs) as described previously by Adnan et al.^64^. A 10-μl aliquot was injected into the SBC18 column (2.1 mm × 50 mm; particle size 1.8 μm). Elution was performed using 1% formic acid in deionized water (solvent A) and acetonitrile (solvent B) at a flow rate of 0.350 ml/min. MS detection was performed using MS Q-TOF in positive and negative atmospheric pressure chemical ionization modes. PubChem was employed as the main tool for the identification of the phytochemical constituents in the sample. Few molecules identified through HRLCMS from the extract were selected based on reports of their antimicrobial activity by other researchers.

### Binding affinity calculation through CB-Dock

The sequence of Big Mitogen-activated Protein Kinase 1 (BMK-1) of *Bipolaris oryzae* was retrieved from UniProt under accession number Q68A24^65^. The model was generated using *I-TASSER* (Iterative Threading ASSEmbly Refinement)^66^. Among the various models, the most appropriate model was chosen. The quality of the model was assessed through PROCHECK software. We selected the model based on the best stereochemical quality evaluated through a Ramachandran plot. As this model manifested 97.1% of the residues in the allowed regions of this plot and only 2.9% of the residues in the disallowed regions, we prioritized this model over others, which demonstrated relatively poor stereochemical quality. The active site residues of BMK-1 were predicted using COACH^67^. Next, the ligands obtained from the methanol extracts of *Syzygium aromaticum* and *Inula racemosa* were retrieved from the large PubChem database^68^. Finally, molecular docking was performed using CB-DOCK2, which is a modified version of CB-DOCK, and the molecules were individually docked against the modeled BMK1 receptor, and binding free energy values were recorded^69^.

## Conclusion

It can be concluded that among the ten plant extracts, clove extracts (methanol and acetone) showed promising results, followed by *Inula racemosa* root extract (methanol) against *B. oryzae*. Acetone was found to be the most efficient among all three extraction solvents used. Among the five molecules found in methanol clove extract, ursolic acid has the highest binding potency against BMK-1, an important enzyme for the growth, conidiation and pathogenicity of *Bipolaris oryzae*. Noscapine from *Inula racemosa* root extract showed maximum binding affinity with BMK-1. These findings conclusively indicate that *Syzygium aromaticum* (clove) bud extract and *Inula racemosa* root extract are sources of a variety of phyto-chemicals that can act as lead compounds for the development of novel fungicides.

### Supplementary Information

Supplementary Tables.

## Data Availability

The datasets generated during and/or analyzed during the current study are available in the supplementary information files.
