# Supplementary material for: Identification of plant based potential antifungal compounds against BMK-1 protein of Bipolaris oryzae using molecular docking approach
Source: Sci Rep. 2024 Jul 8;14:15665. doi: 10.1038/s41598-024-61431-6 (PMC11231321; doi:10.1038/s41598-024-61431-6)
Supplement: Supplementary file 1 — Supplementary Tables. [file 41598_2024_61431_MOESM1_ESM.docx]

**Suplementary Table 1: List of Plants and Plant parts used**

| **SS. No.** | **Scientific name** | **Common name/Local name** | **Plant parts used** | **Voucher/ Specimen No** |
| --- | --- | --- | --- | --- |
| 01 | Syzygium aromaticum | Clove/Rong | Floral buds | 9147-KASH |
| 02 | Sassurea costus | Indian costus/Kuth | Root | 9140-KASH |
| 03 | Acorus calamus | Sweet flag/Vaigunder | Rhizome and leaves | 9146-KASH |
| 04 | Bergenia ciliate | Pal-pout/Zakhmi-Hayat | Roots | 9142-KASH |
| 05 | Geranium pratense | Meadow carnes-bill /Ringresh | Whole plant | 9144-KASH |
| 06 | Mentha longifolia | Mint/pudna | Leaves | 9143-KASH |
| 07 | Inula racemosa | Oriss root/Puskarmool | Roots | 9145-KASH |
| 08 | Podophyllum hexandrum | Wan-wangun/Bankakri | Roots | 9139-KASH |
| 09 | Heracleum candicans | White leaf Hogweed | Leaves | 9141-KASH |
| 110 | Picrorhiza kurroa | Katuka/Kutki | Stem | 9138-KASH |

**Supplementary Table 2: Per cent Mycelial Growth Inhibition of *Bipolaris oryzae* by different concentrations of test plant extracts**

| **Solvents Extracts** | **Methanol** | | | **Sub Mean** | **Acetone** | | | **Sub Mean** | **DMSO** | | | **Sub Mean** | **Factor Means** | | |
| --- | --- | --- | --- | --- | --- | --- | --- | --- | --- | --- | --- | --- | --- | --- | --- |
|  | **C1** | **C2** | **C3** |  | **C1** | **C2** | **C3** |  | **C1** | **C2** | **C3** |  | **Extracts** | **Concentration** |  |
| *Geranium pratense* | 12.22  (3.49) * | 17.22  (4.14) | 26.66  (5.16) | 18.70  (5.16) | 37.50  (6.12) | 47.92  (6.92) | 53.65  (7.32) | 46.35  (6.81) | 0  (0.71) | 0  (0.71) | 3.66  (2.04) | 1.22  (1.15) | 22.09  (4.70^d^) | 34.12  (5.84^c^)  37.82  (6.15^b^)  42.33  (6.50^a^) |  |
| *Inula racemosa* | 80.55  (8.97) | 88.33  (9.39) | 90.33  (9.50) | 86.40  (9.29) | 74.33  (8.62) | 79.67  (8.92) | 81.67  (9.03) | 78.56  (8.86) | 0.36  (0.93) | 0.9  (1.18) | 2.73  (1.80) | 1.33  (1.35) | 55.43  (7.44^b^) |  |  |
| *Acorus calamus* | 42.22  (6.49) | 47.22  (6.87) | 64.44  (8.02) | 51.29  (7.16) | 74.12  (8.60) | 75.88  (8.71) | 81.14  (9.00) | 77.05  (8.78) | 0  (0.71) | 0.36  (0.93) | 2.00  (1.58) | 0.78  (1.13) | 43.04  (6.56^c^) |  |  |
| *Syzygium aromaticum* | 100.00  (10) | 100.00  (10) | 100.00  (10) | 100.00  (10) | 100.00  (10) | 100.00  (10) | 100.0  (10) | 100.00  (10.00) | 4.56  (2.25) | 7.36  (2.80) | 10.17  (3.19) | 7.36  (2.80) | 69.12  (8.31^a^) |  |  |
| *Bergenia ciliata* | 52.78  (7.26) | 54.33  (7.37) | 58.33  (7.63) | 55.14  (7.42) | 0.00  (0.70) | 2.33  (1.68) | 5.00  (2.34) | 2.44  (1.72) | 0  (0.71) | 0  (0.71) | 0  (0.71) | 0  (0.71) | 19.19  (4.38^e^) |  |  |
| *Heracleum candicans* | 61.11  (7.81) | 67.22  (8.19) | 73.33  (8.56) | 67.22  (8.19) | 36.11  (6.00) | 45.56  (6.74) | 61.11  (7.81) | 47.59  (6.90) | 0  (0.71) | 0  (0.71) | 2.38  (1.70) | 0.79  (1.13) | 38.53  (6.20^c^) |  |  |
| *Mentha longifolia* | 0.00  (0.70) | 0.00  (0.70) | 0.00  (0.70) | 0.00  (0.70) | 42.19  (6.49) | 50.52  (7.10) | 54.69  (7.39) | 49.13  (7.01) | 0  (0.71) | 1.26  (1.33) | 2.36  (1.69) | 1.21  (1.30) | 16.78  (4.09^e^) |  |  |
| *Saussurea costus* | 68.33  (8.26) | 74.33  (8.62) | 83.33  (9.12) | 75.33  (8.67) | 61.46  (7.83) | 65.10  (8.06) | 73.44  (8.56) | 66.67  (8.16) | 4.56  (2.25) | 9.23  (3.12) | 13.26  (3.64) | 9.02  (3.08) | 50.34  (7.09^b^) |  |  |
| *Podophyllum hexandrum* | 26.11  (5.10) | 33.33  (5.77) | 36.11  (6.00) | 31.85  (5.64) | 45.56  (6.74) | 52.22  (7.22) | 63.89  (7.99) | 53.89  (7.34) | 3.46  (1.99) | 4.56  (2.25) | 7.4  (2.81) | 5.14  (2.37 | 30.29  (5.50^c^) |  |  |
| *Picrorhiza kurroa* | 36.11  (6.00) | 40  (6.32) | 43.88  (6.62) | 40  (6.32) | 60.00  (7.74) | 68.33  (8.26) | 72.78  (8.53) | 67.04  (8.19) | 0  (0.71) | 1.53  (1.23) | 2.36  (1.53) | 1.30  (1.15) | 36.11  (6.00^c^) |  |  |
| Mean | 47.94  (6.92) | 52.20  (7.22) | 57.64  (7.59) | 52.59  (7.25^b^) | 53.13  (7.28) | 58.75  (7.66) | 64.74  (8.04) | 58.87  (7.67^a^) | 1.29  (1.13) | 2.52  (1.58) | 4.63  (2.15) | 2.81  (1.62^c^) |  |  |  |
| **CD (p<0.05)** | Extracts (E) = 0.81 Solvent (S) = 0.44 Concentration (C) = 0.33  Extracts × Solvent (E*S) =1.41 Extracts × Concentration (E*C) = 1.41 Solvent*Concentration (S*C) = 0.77  Extracts*Solvent*Concentration (E*S*C) = 2.44 | | | | | | | | | | | | | | |

**Supplementary Table 3 :** **Spore Germination inhibition of *Bipolaris oryzae* by different concentrations of test plant extracts**

| **Solvents**  **Extracts** | **Methanol** | | | **Sub Mean** | **Acetone** | | | **Sub Mean** | **DMSO** | | | **Sub Mean** | **Factor Means** | |
| --- | --- | --- | --- | --- | --- | --- | --- | --- | --- | --- | --- | --- | --- | --- |
|  | **C1** | **C2** | **C3** |  | **C1** | **C2** | **C3** |  | **C1** | **C2** | **C3** |  | **Extracts** | **Concentration** |
| *Geranium*  *pratense* | 35.80  (5.88) | 54.32  (7.36) | 64.2  (8.01) | 51.44  (7.08) | 11.67  (3.33) | 28.33  (5.32) | 46.67  (6.83) | 41.78  (5.16) | 2.50  (1.73) | 3.50  (2.00) | 5.67  (2.48) | 3.89  (2.09) | 32.37  (5.68^g^) | 30.47  (5.51^c^)  45.37  (6.73^b^)  53.61  (7.32^a^) |
| *Inula*  *racemosa* | 48.15  (6.94) | 60.49  (7.77) | 76.54  (8.74) | 61.73  (7.82) | 58.33  (7.63) | 76.67  (8.75) | 86.67  (9.31) | 66.94  (8.56) | 2.20  (1.64) | 2.53  (1.74) | 3.17  (1.91) | 2.63  (1.77) | 43.77  (6.61^cd^) |  |
| *Acorus*  *Calamus* | 22.22  (4.68) | 66.67  (8.16) | 79.01  (8.89) | 55.97  (7.24) | 26.67  (5.16) | 55.00  (7.40) | 76.67  (8.75) | 54.60  (7.10) | 5.50  (2.44) | 5.67  (2.48) | 5.83  (2.51) | 5.67  (2.48) | 38.74  (6.22^de^) |  |
| *Syzygium aromaticum* | 43.7  (6.60) | 66.67  (8.13) | 58.52  (7.62) | 56.30  (7.45) | 51.67  (7.18) | 76.67  (8.75) | 86.67  (9.31) | 62.89  (8.41) | 6.70  (2.68) | 7.83  (2.88) | 8.50  (3.00) | 7.68  (2.85) | 42.29  (6.50^bc^) |  |
| *Bergenia*  *ciliata* | 20.74  (4.41) | 59.26  (7.69) | 70.37  (8.38) | 50.12  (6.83) | 20.00  (4.32) | 48.33  (6.94) | 68.33  (8.27) | 48.16  (6.51) | 2.50  (1.73) | 3.33  (1.95) | 6.50  (2.64) | 4.11  (2.14) | 34.13  (5.84^fg^) |  |
| *Heracleum candicans* | 76.54  (8.75) | 82.72  (9.09) | 91.36  (9.56) | 83.54  (9.13) | 66.67  (8.16) | 81.67  (9.04) | 91.67  (9.57) | 82.02  (8.92) | 5.70  (2.48) | 6.43  (2.63) | 7.00  (2.73) | 6.38  (2.62) | 57.31  (7.57a) |  |
| *Mentha*  *longifolia* | 43.59  (6.56) | 66.67  (8.16) | 79.49  (8.91) | 63.25  (7.88) | 21.67  (4.65) | 36.67  (6.04) | 48.33  (6.95) | 51.38  (5.88) | 2.67  (1.77) | 7.50  (2.82) | 9.33  (3.13) | 6.50  (2.64) | 40.38  (6.35^ab^) |  |
| *Saussurea*  *costus* | 56.79  (7.53) | 70.37  (8.39) | 79.01  (8.89) | 68.72  (8.27) | 46.67  (6.76) | 66.67  (8.16) | 78.33  (8.85) | 66.65  (7.92) | 10.17  (3.18) | 11.60  (3.40) | 12.30  (3.57) | 11.36  (3.36) | 48.91  (6.99^a^) |  |
| *Podophyllum hexandrum* | 44.87  (6.65) | 76.92  (8.76) | 84.62  (9.19) | 68.80  (8.20) | 66.67  (8.16) | 76.67  (8.75) | 91.67  (9.57) | 72.89  (8.83) | 11.11  (3.33) | 12.37  (3.51) | 13.67  (3.69) | 12.38  (3.51) | 51.36  (7.16^a^) |  |
| *Picrorhiza*  *kurroa* | 55.56  (7.43) | 71.6  (8.46) | 82.72  (9.09) | 69.96  (8.33) | 41.67  (6.31) | 66.67  (8.16) | 81.67  (9.03) | 67.12  (7.83) | 5.50  (2.44) | 11.41  (3.37) | 13.80  (3.78) | 10.24  (3.19) | 49.11  (7.00^abc^) |  |
| Mean | 44.80  (6.54) | 67.57  (8.20) | 76.58  (8.73) | 62.98  (7.82^a^) | 41.17  (6.17) | 61.34  (7.73) | 75.67  (8.64) | 61.44  (7.51^b^) | 5.45  (2.44) | 7.22  (2.77) | 8.58  (3.01) | 7.08  (2.75^c^) |  |  |
| **CD (p<0.05)** | Extracts (E) = 0.46 Solvent (S) = 0.25 Concentration (C) = 0.25  Extracts × Solvent (E*S) = 0.80 Extracts × Concentration (E*C) = 0.80 Solvent*Concentration (S*C) = 0.43  Extracts*Solvent*Concentration (E*S*C) = 1.38 | | | | | | | | | | | | | |

*Values in parenthesis are square root transformed value, C1: 2000ppm C2: 3000ppm C3: 4000ppm

**Supplementary Table 4; List of compounds detected in methanolic flower bud extract of *Syzygium aromaticum***

| **S. No.** | **Name of the Compound** | **Class** |
| --- | --- | --- |
| 1 | Eugenol | Phenolic compound |
| 2 | Quercetin | Flavonoid |
| 3 | Chlorogenic acid | Phenolic acid |
| 4 | Gallic acid | Poly-phenol |
| 6 | Rhein / Cassic acid | Anthraquinone |
| 7 | Ursolic acid | Tri- terpene |
| 9 | Aesculin | Coumarin glucoside |
| 11 | Lecanoric acid | Poly- phenol |
| 12 | Rhamnetin | Flavanoid |
| 13 | Ellagic acid | Poly- phenol |
| 15 | Luteoline | Flavanoid |
| 16 | Diosmin | Flavone glycoside |
| 17 | Cefuroxime axetil | β- lactam antibiotic |
| 18 | Koparin | Flavonoid |
| 19 | Geneticin | Aminoglycoside antibiotic |
| 20 | Norstictic acid | Depsidone |

**Supplementary Table 5: List of compounds detected in methanolic root extract of *Inula racemosa***

| **S. No.** | **Name of the Compound** | **Class** |
| --- | --- | --- |
| 01 | Quinic acid | Cyclic polyol |
| 02 | Dihydroisoalantolactone | Eudesmanolides |
| 03 | m-Coumaric acid | Hydroxycinnamic acid |
| 04 | Caffeic acid | Hydroxycinnamic acid |
| 05 | Isoferullic acid | Hydroxycinnamic acid |
| 06 | Ellagic acid | Hydrolyzable tannins |
| 07 | Cryptochlorogenic acid | Cyclic polyol |
| 08 | Myricetin 7-rhamnoside | Flavonol |
| 09 | Osmanthuside A | Cinnamic acid |
| 10 | Glaucarubin | Quassinoid |
| 11 | Sanggenon C | Flavonoid |
| 12 | Apotrichodiol | Tetrahyrdofurans |
| 13 | Noscapine | Alkaloid |
| 14 | Cammaconine | Diterpenoid |
| 15 | Istamycin C1 | Formamide |
| 16 | Thalicsessine | Diterpenoid |
| 17 | Mosesin 4 | Steroid Saponin |
| 18 | Pinguisone | Sesquiterpenoid |
| 19 | Moracin | Benzofuran |

**Supplementary Table 6: Binding affinity values of different molecules identified from methanolic extract of *Syzygium aromaticum* and the BMK-1 residues interacting with them**

| **Name of Molecule** | **Target Enzyme** | **Binding Affinity (kcal/mol)** | **Contact Residues** |
| --- | --- | --- | --- |
| Quercetin |  | −8.1 | VAL26, TYR31, VAL34, ALA47, LYS49, ARG62, GLU101, LEU102, MET103,GLU104,THR105, ASP106, SER148,ASN149, LEU151, ASP162 |
| Eugenol | BMK-1 | −5.2 | VAL26, VAL34, ALA47, LYS49, GLU66, ILE79, GLN100, LEU102, MET103, SER148, LEU151, CYS161, ASP162 |
| Chlorogenic acid |  | −8.0 | VAL26, GLY27, GLU28, GLY29,TYR31, GLY32, VAL33, VAL34, ALA47, LYS49, GLU66, ILE79, GLN100, GLU101, LEU102, MET103, GLU104, THR105, ASP106, LYS146, SER148, ASN149, LEU151, CYS161, ASP162 |
| Gallic acid |  | −5.0 | VAL34, ALA47, LYS49, GLU66, ILE79, GLN100, GLU101, SER148,LEU151, CYS161, ASP162 |
| Ursolic acid |  | −8.7 | VAL26, GLY27, GLU28, GLY29, ALA30, TYR31, GLY32, VAL33, VAL34, ALA47, LYS49, ILE79, LEU102, MET103, GLU104, THR105, ASP106, SER148, ASN149, LEU151, CYS161, ASP162 |

**Supplementary Table 7: *Inula racemosa* molecules and their binding affinity and interaction profile in docked state with BMK-1.**

| **Molecule** | **Receptor Enzyme** | **Binding Affinity (kcal/mol)** | **Residues in contact** |
| --- | --- | --- | --- |
| Quinic acid | BMK-1 | −5.1 | VAL26 GLY27 GLU28 GLY29 ALA30 TYR31 GLY32 VAL33 VAL34 LYS49 ARG62 SER148 ASN149 LEU151 CYS161 ASP162 |
| M-Coumaric acid |  | −5.6 | VAL26 VAL34 ALA47 LYS49 ILE79 GLN100 GLU101 LEU102 MET103 GLU104 SER148 LEU151 CYS161 ASP162 |
| Caffeic acid |  | −5.2 | VAL26 VAL34 LYS49 GLU66 ILE79 GLN100 ASP106 SER148 LEU151 CYS161 ASP162 |
| Cryptochlorogenic acid |  | −7.6 | VAL26 GLY27 GLU28 GLY29 TYR31 GLY32 VAL34 ALA47 LYS49 GLU66 ILE79 GLN100 GLU101 LEU102 MET103 GLU104 THR105 SER148 ASN149 LEU151 CYS161 ASP162 |
| Noscapine |  | −8.1 | VAL26 GLY27 GLU28 GLY29 ALA30 TYR31 GLY32 VAL34 ALA47 LYS49 ARG62 ILE79 GLN100 GLU101 MET103 ASP106 ASP144 LYS146 SER148 ASN149 LEU151 CYS161 ASP162 |
